# The transcription factor Vezf1 represses the expression of the antiangiogenic factor Cited2 in endothelial cells

**DOI:** 10.1074/jbc.RA118.002911

**Published:** 2018-05-24

**Authors:** Lama AlAbdi, Ming He, Qianyi Yang, Allison B. Norvil, Humaira Gowher

**Affiliations:** From the ‡ Department of Biochemistry and; § Purdue University Center for Cancer Research, Purdue University, West Lafayette, Indiana 47907

**Keywords:** embryonic stem cell, endothelial cell, cell differentiation, E1A-binding protein p300 (P300), gene regulation, chromatin immunoprecipitation (ChiP), DNA methylation, histone acetylation, vascular endothelial growth factor (VEGF), angiogenesis, Cited2, H3K27 acetylation, Vezf1

## Abstract

Formation of the vasculature by angiogenesis is critical for proper development, but angiogenesis also contributes to the pathogenesis of various disorders, including cancer and cardiovascular diseases. Vascular endothelial zinc finger 1 (Vezf1), is a Krüppel-like zinc finger protein that plays a vital role in vascular development. However, the mechanism by which Vezf1 regulates this process is not fully understood. Here, we show that *Vezf1*^−/−^ mouse embryonic stem cells (ESC) have significantly increased expression of a stem cell factor, Cbp/p300-interacting transactivator 2 (Cited2). Compared with WT ESCs, *Vezf1*^−/−^ ESCs inefficiently differentiated into endothelial cells (ECs), which exhibited defects in the tube-formation assay. These defects were due to reduced activation of EC-specific genes concomitant with lower enrichment of histone 3 acetylation at Lys^27^ (H3K27) at their promoters. We hypothesized that overexpression of Cited2 in *Vezf1*^−/−^ cells sequesters P300/CBP away from the promoters of proangiogenic genes and thereby contributes to defective angiogenesis in these cells. This idea was supported by the observation that shRNA-mediated depletion of Cited2 significantly reduces the angiogenic defects in the *Vezf1*^−/−^ ECs. In contrast to previous studies that have focused on the role of Vezf1 as a transcriptional activator of proangiogenic genes, our findings have revealed a role for Vezf1 in modulating the expression of the antiangiogenic factor Cited2. Vezf1 previously has been characterized as an insulator protein, and our results now provide insights into the mechanism, indicating that Vezf1 can block inappropriate, nonspecific interactions of promoters with *cis*-located enhancers, preventing aberrant promoter activation.

## Introduction

Development of a proper vascular system is indispensable for embryogenesis. Accurate spatial and temporal control of gene expression is required in endothelial cells (ECs),^4^ which are committed to the formation of the vasculature (1, 2). Angiogenesis involves migration, growth, and differentiation of ECs and takes place during development as well as in adulthood. Angiogenesis is regulated by an interplay between pro- and anti-angiogenic factors (3). Hif-1α is a major pro-angiogenic factor, which interacts with p300/CBP and activates the expression of a number of pro-angiogenic genes including *VEGF*, thus initiating new vessel formation. Treatment of cultured ECs with VEGF-A_165_ induces Hif-1α expression, suggesting a bidirectional stimulatory relationship between VEGF and Hif-1α (4). *In vivo*, *Hif-1*α can be induced by a variety of factors including hypoxic conditions, (5–7), and certain cytokines and growth factors under normoxic conditions (8–12). Among many known factors, Vezf1 (DB1/Bgp1) and Cited2 (Mrg1/p35srj) play important roles in regulation of angiogenesis during development and in adulthood.

Cited2 (Cbp/p300-interacting transactivator with Glu/Asp-rich carboxyl-terminal domain 2) also named Mrg1/p35srj is a ubiquitously expressed essential transcriptional regulator that binds strongly to the histone acetyltransferases p300 and CBP (cAMP-responsive element-binding protein). Cited2 plays a critical role in heart development, neurulation, and maintenance of fetal and adult hematopoietic stem cells. It is expressed throughout early embryogenesis and in embryonic stem cells (ESCs) (13–17, 19–21). Several studies have demonstrated that by competing with Hif-1α to bind CBP/P300, Cited2 prevents the activation of pro-angiogenic genes such as *VEGF*, and inhibits angiogenesis (22–24). For example, over-expression of Cited2 suppresses *VEGF* promoter activity, and siRNA knockdown of Cited2 increases *VEGF* promoter activity (25). The expression levels of Hif-1α responsive genes including VEGF is increased in Cited2^−/−^ embryos (14). Another study showed that Cited2 is a negative regulator of fracture healing, and its expression is inversely related to the expression of genes involved in extracellular matrix remodeling and angiogenesis, such as matrix metalloprotease, VEGF, and Hif-1α (26). These studies suggest that the proper up-regulation of pro-angiogenic genes requires the levels of Cited2 to be tightly controlled especially at the onset of angiogenesis. Because its aberrant high expression in a specific developmental window can inhibit Hif-1α activity, this regulation can potentially be critical for angiogenesis in both embryonic stages and adulthood.

Mammalian Vezf1 is an essential transcription factor, which is expressed in the anterior-most mesoderm at E7.5 during development. Its expression is later restricted in the vascular endothelium, an observation that revealed its role in regulation of angiogenesis. *Vezf1* null mice die at approximately E9.5 (27). *Vezf1*^−/−^ ESCs grow slower and make smaller embryoid bodies, which have defects in vascularization and cease to grow a few days post-differentiation (28, 29). *Vezf1* is expressed in both adult and embryonic ECs. Blocking the activity of Vezf1 by small molecule inhibitor Vec6 inhibits wound healing suggesting its role in postnatal angiogenesis (30). Vezf1 contains six Cys_2_/His_2_-type zinc finger motifs and binds poly(dG) or poly(dC) sequences (31, 32). It carries a glutamine-rich stretch and a proline-rich region that are characteristic of transcriptional activation or repression domains (33). It is proposed to act as a transcriptional activator of pro-angiogeneic genes including *endothelin 1*, *microtubule turnover protein*, *stathmin*/*OP18*, and *metallothionein* 1 (*MT1*) (34–36). However, no change in the expression of pro-angiogenic genes was seen in *Vezf1*^−/−^ embryos (28). Other studies suggested an indirect role of Vezf1 by interacting with RhoB that promotes expression of RhoB-regulated pro-angiogenesis genes (30, 37). Therefore, the mechanism by which Vezf1 regulates angiogenesis is unclear.

To study the specific role of Vezf1 in endothelial development and angiogenesis, we examined the differentiation of WT and *Vezf1*^−/−^ ESCs to ECs by treatment with VEGF-A_165_ and tested their angiogenic potential by a *in vitro* tube-formation assay. Our findings suggest that Vezf1 controls activation of angiogenesis in ECs by restricting *Cited2* expression to basal levels, which allows Hif-1α–mediated activation of the pro-angiogenic genes. We observed a strong increase in the expression of *Cited2* in *Vezf1*^−/−^ ESCs compared with WT cells. In addition, the elevated expression of *Cited2* in *Vezf1*^−/−^ ESCs affected their efficiency of differentiation into ECs and attenuated the ability of *Vezf1*^−/−^ ECs to form vascular structures in a tube-formation assay. Concomitant with this, there was reduced activation of endothelial/pro-angiogenic genes in differentiating Vezf1^−/−^ ECs. Based on our data showing reduced levels of histone H3K27Ac at the promoters of angiogenesis-specific genes, we propose that high levels of Cited2 sequester the histone acetyltransferase p300 away from angiogenesis-specific gene promoters, thus reducing their activation and gene expression. Together these observations substantiate the critical role of Vezf1 in controlling the expression of developmental regulators such as Cited2. Given that the expression of *Cited2* in ESCs is not completely turned off, we suggest the role of Vezf1 in fine-tuning *Cited2* expression in ESCs. Our previous work using genome-wide ChIP-SEQ showed that binding sites for Vezf1 are mostly present in CpG-rich regions (38). We also showed that Vezf1 binds to the chicken β-globin insulator suggesting a role in regulating enhancer-mediated control of gene expression (32, 38). Based on these studies, we speculate that the insulator function of Vezf1 blocks inappropriate interactions of the *Cited2* promoter with nearby enhancer/s, thus modulating the magnitude and spatiotemporal regulation of its expression.

## Results

### Cited2 expression is high in Vezf1^−/−^ ESCs

**Figure 1.**
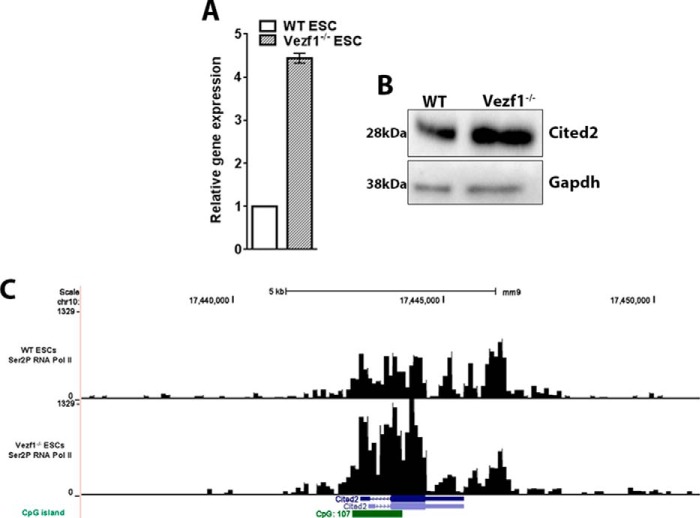
*A,* gene expression analysis of *Cited2* by RT-qPCR in WT and *Vezf1*^−/−^ ESCs showed a 4–5-fold higher expression of *Cited2* in *Vezf1*^−/−^ ESCs. Gene expression was normalized to Gapdh and represented relative to gene expression in WT ESC, set to 1. *B*, 50 μg of total cell extract were loaded from the WT and *Vezf1*^−/−^ ESCs followed by Western blot analysis using anti-Cited2 antibody and anti-GAPDH antibody for loading control. *C,* screen shot from UCSC genome browser showing the occupancy of Ser^2^-phosphorylated Pol II at the Cited2 locus in WT and *Vezf1*^−/−^ ESCs. These data were obtained from the previously published publically available ChIP-SEQ data. *WT*, wildtype ESCs; *Vezf1*^−/−^, Vezf1-knockout ESCs.

To elucidate the mechanism of Vezf1, we had previously analyzed changes in gene expression of Vezf1^−/−^ ESCs compared with WT ESCs using a microarray analysis (Fig. S1) (39). We found *Cited2* among the top 20 genes that were up-regulated in *Vezf1*^−/−^ ESCs by more than 5-fold. To confirm this observation, we measured the gene expression of *Cited2* quantitatively in WT and *Vezf1*^−/−^ ESCs by qRT-PCR and protein levels by Western blotting. The data show a 4–5-fold higher transcript and protein levels of Cited2 in *Vezf1*^−/−^ ESCs compared with WT ESCs (Fig. 1, *A* and *B*). We found these observations consistent with our previously published ChIP-SEQ of Ser^2^ phosphorylated RNA Pol II (elongating form of RNA Pol II) in WT and *Vezf1*^−/−^ ESCs (38). Data analysis of elongating RNA Pol II showed more than a 2-fold higher enrichment in the Cited2 gene body in *Vezf1*^−/−^ ESCs compared with the WT ESCs (Fig. 1*C*). Based on the known function of Cited2 as an anti-angiogenic factor (22–24), we hypothesized that a presence of high Cited2 level in *Vezf1*^−/−^ cells could interrupt or delay the differentiation of ECs, or reduce their angiogenic potential.

### Vezf1^−/−^ ESCs are defective in EC differentiation

**Figure 2.**
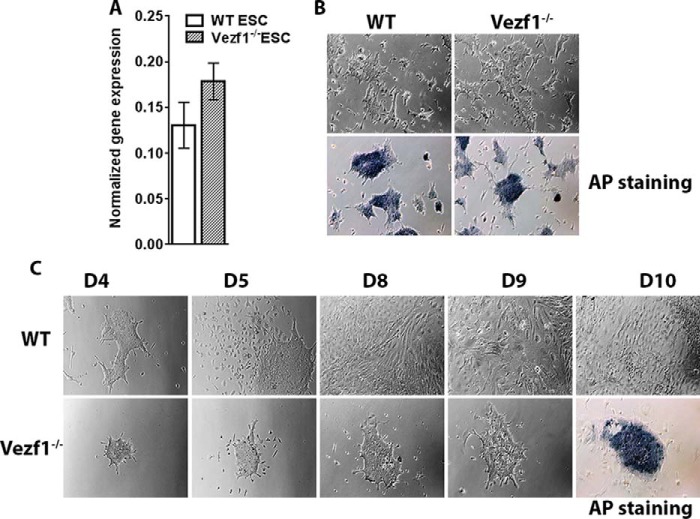
*A,* gene expression analysis by RT-qPCR of Oct4 in WT and *Vezf1*^−/−^ ESCs. *B,* alkaline phosphatase (*AP*) staining for pluripotency in WT and *Vezf1*^−/−^ ESCs; the presence of *dark blue* stain indicates positive for pluripotency. *C*, WT and *Vezf1*^−/−^ ESCs were differentiated using 10 ng/μl of VEGF-A_165_ for 5, 6, 7, and 10 days and visualized using brightfield microscopy at ×10 magnification. Unlike the WT cells, the *Vezf1*^−/−^ cells were unable to differentiate and most of the cells died. The field view is the representation of proliferation during differentiation and cell number. The panel for D10 shows alkaline phosphatase staining. A strong signal of *Vezf1*^−/−^ cells indicates presence of undifferentiated stem cells. *WT*, wildtype ESCs; *Vezf1*^−/−^, *Vezf1* knockout ESCs; *UD*, undifferentiated; D4–D10, days post-differentiation.

*Cited2* expression is critical for pluripotency and differentiation of ESCs; therefore, we tested if overexpression of Cited2 affected the pluripotency of *Vezf1*^−/−^ ESCs. We quantified the expression of the pioneer transcription factor, Oct4, in *Vezf1*^−/−^ ESCs compared with that in the WT ESCs and observed no significant difference in its expression (Fig. 2*A*). Additionally, both WT and Vezf1^−/−^ ESCs showed positive alkaline phosphate staining suggesting that high expression of Cited2 had little if any effect on the pluripotency of *Vezf1*^−/−^ ESCs (Fig. 2*B*). Previous studies showed a reduced growth of *Vezf1*^−/−^ EBs and defects in their vascular structures, but reported little or no difference in the endothelial differentiation in 3D cultures (27). We performed *in vitro* differentiation of WT and *Vezf1*^−/−^ ESCs to ECs on gelatinized plates in the presence of 10 ng/μl of VEGF-A_165_. The differentiation of ESCs to ECs was monitored by microscopy. During differentiation, the WT ESCs showed an expected loss of ∼5–10% of cells, and differentiated efficiently into ECs. Comparatively, during the first 3 days of differentiation, over 80% *Vezf1*^−/−^ cells died, leading to a reduced efficiency of EC derivation from the *Vezf1*^−/−^ ESCs. This was confirmed by positive alkaline phosphatase staining of the surviving *Vezf1*^−/−^ cells at 10 days post-differentiation (Fig. 2*C*).

**Figure 3.**
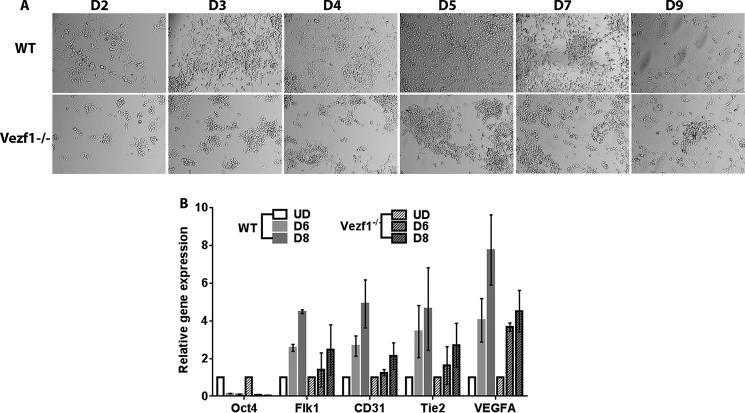
*A* and *B,* WT and *Vezf1*^−/−^ ESCs were differentiated using 20 ng/μl of VEGF-A_165_. *A,* differentiating WT and *Vezf1*^−/−^ ECs were visualized using brightfield microscopy. Whereas most of the WT cells undergo distinct morphological changes, only a fraction acquire a similar morphology in *Vezf1*^−/−^ cells. *B,* gene expression by RT-qPCR plotted as a relative change to the expression in UD where UD was set to 1. Endothelial specific genes, *Flk1*, *VEGF-A*, *CD31*, and *Tie2* show an expected increase in expression in differentiating WT ECs. Differentiating *Vezf1*^−/−^ ECs, however, show significantly low expression of all tested endothelial-specific genes. A decrease in Oct4 expression is observed in both WT and *Vezf1*^−/−^ ECs indicating loss of pluripotency. *WT*, wildtype; *Vezf1*^−/−^, *Vezf1* knockout; *UD*, undifferentiated; *D2–D9*, days post-differentiation.

**Figure 4.**
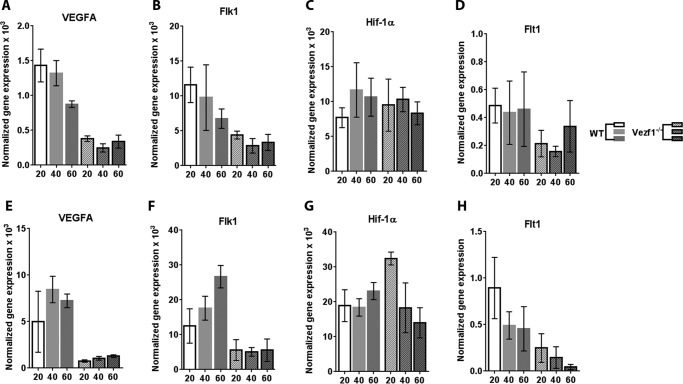
*A–D,* gene expression analysis in WT and *Vezf1*^−/−^ D6 ECs, differentiated using 20, 40, and 60 ng/μl of VEGF-A_165_. *E–H*, gene expression analysis in WT and *Vezf1*^−/−^ D8 ECs differentiated using 20, 40 and 60 ng/μl of VEGF-A_165_. The expression of all genes is increased in D8 compared with D6 post-differentiation. Higher doses of VEGF-A_165_ have no further effect on expression of VEGF-A, Flk1, Hif-1α, and Flt1 in both WT or in *Vezf1*^−/−^ cells. The data represents average and S.D. of 3 to 4 replicates. *WT*, wildtype; *Vezf1*^−/−^, *Vezf1* knockout; *UD*, 20, 40, 60 ng/μl of VEGF-A_165_ used for differentiation.

Given that the expression of the EC lineage is driven by VEGF-A in an autoregulatory loop (9, 40), we tested if increasing the concentration of VEGF-A_165_ in the medium could improve EC differentiation and survival of *Vezf1*^−/−^ cells. EC differentiation was monitored by measurement of endothelial-specific gene expression including *VEGF-A*, *Flk1*, *Flt1*, *CD31*, and *Tie2* (41). WT and Vezf1^−/−^ ESCs were differentiated using 20, 40, and 60 ng/μl of VEGF-A_165_. Increasing the VEGF-A_165_ to 20 ng/μl stimulated *Vezf1*^−/−^ cells to differentiate to ECs with a higher efficiency (Fig. 3*A*). In both WT and *Vezf1*^−/−^ ESCs, differentiation induced repression of the pluripotency gene, Oct4, and activation of endothelial-specific genes, *VEGF-A*, *Flk1*, *CD31*, and *Tie2*, albeit at lower levels in the *Vezf1*^−/−^ cells (Fig. 3*B*). Reduced activation of *CD31* and *Tie2* in *Vezf1*^−/−^ cells suggested a partial defect in EC differentiation. WT and *Vezf1*^−/−^ ECs day (D) 6 and D8 post-differentiation treated with 20, 40, and 60 ng/μl of VEGF-A_165_ were collected for gene expression analysis by qRT-PCR. The data showed that at all three concentrations of VEGF-A_165_, the expression of *VEGF-A* and its receptor, Flk1, was significantly higher in WT ECs compared with that in the *Vezf1*^−/−^ ECs, indicating that gene expression is not further rescued by higher doses of VEGF-A_165_ (Fig. 4). Previous studies have shown that *Hif-1*α expression can also be activated by treatment of ECs with VEGF-A_165_ thus showing that VEGF regulates the expression of its own transcription factor (4). In WT and *Vezf1*^−/−^ ECs, we checked the expression of *Hif-1*α and *Flt1*, which is the angiogenesis-specific VEGF receptor. Although no difference in the expression of *Hif-1*α was observed between WT and *Vezf1*^−/−^ ECs, similar to *Flk1* and *VEGF-A*, *Flt1* expression was also comparatively lower in *Vezf1*^−/−^ ECs at 20, 40, and 60 ng/μl of VEGF-A_165_ in both D6 (Fig. 4, *A–D*) and D8 (Fig. 4, *E–H*) post-differentiation. These data show that in *Vezf1*^−/−^ ECs, reduced expression of angiogenic genes including VEGF is not due to lower *Hif-1*α expression, but potentially due to its reduced activity.

### Vezf1^−/−^ ECs are defective in forming vascular networks in 3D cultures

**Figure 5.**
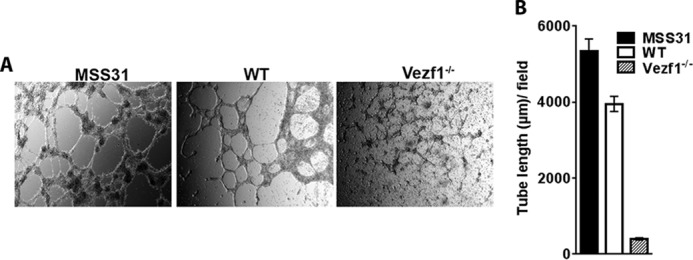
*A,* differentiated WT and *Vezf1*^−/−^ EC plated in VEGF-supplemented Matrigel were incubated at 37 °C for 5–15 h. The formation of tube structures is visualized by brightfield microscopy. Mouse endothelial cell line MSS31 is used as a positive control. The images were taken at ×10 magnification at 12 h for MSS31, and 6 h for WT and *Vezf1*^−/−^. *B,* measurement of tube length using ImageJ software. Compared with MSS31 and WT ECs, *Vezf1*^−/−^ ECs were unable to make tube-like structures in Matrigel. *WT*, wildtype; *Vezf1*^−/−^, *Vezf1* knockout.

We next tested the angiogenic potential of *Vezf1*^−/−^ ECs by an *in vitro* tube-formation assay. We differentiated WT and *Vezf1*^−/−^ ESCs to ECs for 8 days in the presence of VEGF-A_165_ at 20 ng/μl. The ECs were collected and placed in Matrigel to form vascular networks or tubes in 3D culture. The mouse endothelial cell line, MSS31, was used as a positive control. Whereas WT ECs formed distinct tubes within 4–6 h in Matrigel, *Vezf1*^−/−^ ECs showed significant defects in tube formation as indicated by the shorter tube length (Fig. 5, *A* and *B*).

Taken together, these data show that *Vezf1*^−/−^ ESCs have reduced competence to differentiate into ECs and to form vascular structures in Matrigel. Given that Flt1 receptor function is required for tubulogenesis (42), the inability of *Vezf1*^−/−^ ECs to form tubes in Matrigel could be the consequence of strongly reduced expression of *Flt1* in these cells. Because no change was observed in *Hif-1*α gene expression, we predict that the reduced expression of EC-specific genes is due to high *Cited2* expression in *Vezf1*^−/−^ ESCs and ECs.

### Induced repression of Cited2 partially rescues EC differentiation and vascular defects in Vezf1^−/−^ ECs

**Figure 6.**
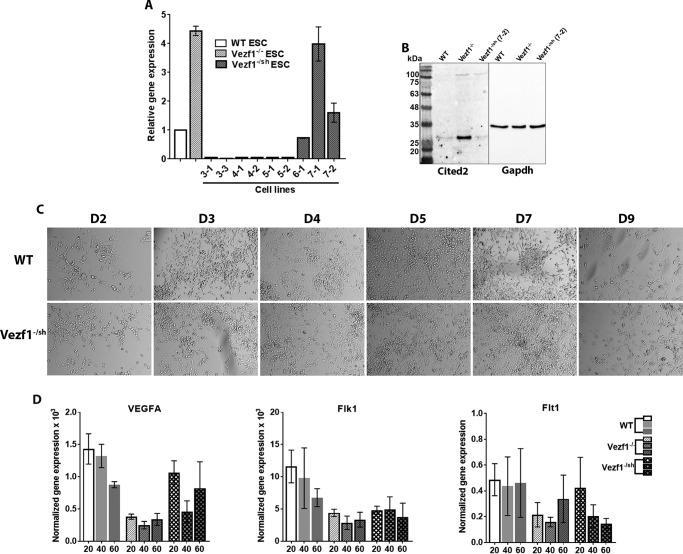
*A,* gene expression analysis of *Cited2* by RT-qPCR in *Vezf1*^−/sh^ cell lines. Change in gene expression was plotted relative to that of WT ESCs, set to 1. Labels on the *x* axis represent various stable cell lines, of which the *Vezf1*^−/sh^ cell line (7-2) has *Cited2* expression reduced to the levels similar to WT ESCs. *B,* Western blot analysis using 50 μg of total cell extract from the WT, *Vezf1*^−/−^, and *Vezf1*^−/sh^ (7-2) ESCs probed with anti-Cited2 antibody and anti-GAPDH. *C,* differentiation of WT, *Vezf1*^−/−^, and *Vezf1*^−/sh^ (7-2) ESCs was induced using 20 ng/μl of VEGF-A_165_. D2–D6 are days post-differentiation. Compared with the differentiating WT cells, *Vezf1*^−/sh^ (7-2) show similar morphology and cell number indicating at least a partial rescue of their ability to differentiate into ECs. *D,* gene expression analysis of *Vezf1*^−/sh^ (7-2) ESCs, which were differentiated to ECs at 20, 40, and 60 ng/μl of VEGF-A_165_. Expression of pro-angiogeneic genes *VEGF-A*, *Flk1*, and *Flt1* was measured. Compared with WT and *Vezf1*^−/−^ cells, gene expression was largely rescued in *Vezf1*^−/sh^ (7-2) ECs. The data represent the average and S.D. of 3 to 4 replicates. *WT,* wildtype ESCs; *Vezf1*^−/−^, Vezf1 knockout ESCs; *Vezf1*^−/sh^, stable transgenic *Vezf1*^−/−^ ESCs expressing Cited2-shRNA; *20*, *40*, *60*, ng/μl of VEGF-A_165_ used for differentiation.

**Figure 7.**
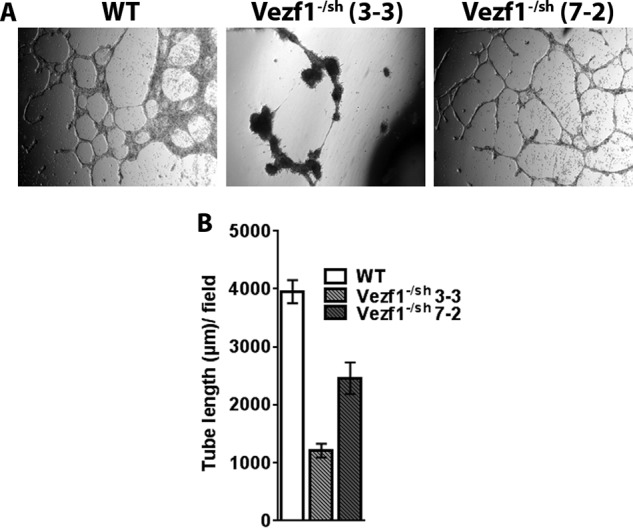
*A,* WT, *Vezf1*^−/sh^ (7-2), and *Vezf1*^−/sh^ (3-3) cells were differentiated for 10 days and used in a tube-formation assay. The *Vezf1*^−/sh^ cell line (3-3) had about 7-fold lower Cited2 expression than WT. Compared with the WT cells, tube formation was rescued in *Vezf1*^−/sh^ (7-2) ECs, which was absent in *Vezf1*^−/sh^ (3-3) ECs. Images were taken at 12 h for *Vezf1*^−/sh^ (3-3) and 6 h for WT and *Vezf1*^−/sh^ (7-2). *B,* tube length was measured by ImageJ software and plotted.

We next tested the hypothesis that the defective vasculature formation by *Vezf1*^−/−^ ECs is due to anomalous high expression of *Cited2*. We therefore asked if depletion of Cited2 in *Vezf1*^−/−^ ESCs could rescue their ability to differentiate and make vascular networks. *Vezf1*^−/−^ ESCs were transfected with *Cited2* shRNA, to generate stable transgenic ESCs lines, *Vezf1*^−/sh^. Of the nine transgenic lines, some showed more than 10-fold reduction in Cited2 expression when compared with WT ESCs. Because Cited2 is known to be important for pluripotency (43), we chose to use the *Vezf1*^−/sh^ cell line (7-2), in which Cited2 expression is reduced to levels similar to that of WT ESC (Fig. 6, *A* and *B*). The *Vezf1*^−/sh^ ESCs were differentiated to ECs using 20, 40, and 60 ng/μl of VEGF-A_165_. Compared with *Vezf1*^−/−^ ESCs, *Vezf1*^−/sh^ ESCs showed better survival and higher efficiency of EC differentiation at 20 ng/μl of VEGF-A_165_, which was similar to the WT cells (Fig. 6*C*). The *Vezf1*^−/sh^-derived ECs were collected on day 6 post-differentiation and RNA was extracted for gene-expression analysis. The data show that expression of VEGF-A was largely rescued, whereas Flt1 and Flk1 were partially rescued when compared with their expression in WT and *Vezf1*^−/−^ ECs (Fig. 6*D*). Next, we tested if the derived ECs form vascular structures by a tube-formation assay. WT, *Vezf1*^−/−^, and *Vezf1*^−/sh^-derived ECs ((7-2) and (3-3)) were collected after 10 days and used to perform an *in vitro* tube-formation assay. *Vezf1*^−/sh^ (3-3) ESCs have ∼7-fold reduced expression of *Cited2* compared with WT ESCs. This cell line was therefore used to test the effect of Cited2 deficiency on EC differentiation and tube formation. The repression of *Cited2* in *Vezf1*^−/sh^ ECs largely rescued the defective tube formation on Matrigel, which was more prominent in *Vezf1*^−/sh^ (7-2) compared with the (3-3) cell line (Fig. 7, *A* and *B*). This observation supports the known role of Cited2 in pluripotency and differentiation of ESCs and emphasizes the importance of the appropriate levels of developmental transcription factors for proper differentiation. These data directly support our hypothesis that an aberrant high expression of *Cited2* prevents the activation of EC-specific gene expression potentially by sequestering p300 from the promoters of angiogenesis-specific genes.

### P300 activity is regulated by Cited2 at the VEGF-A promoter

**Figure 8.**
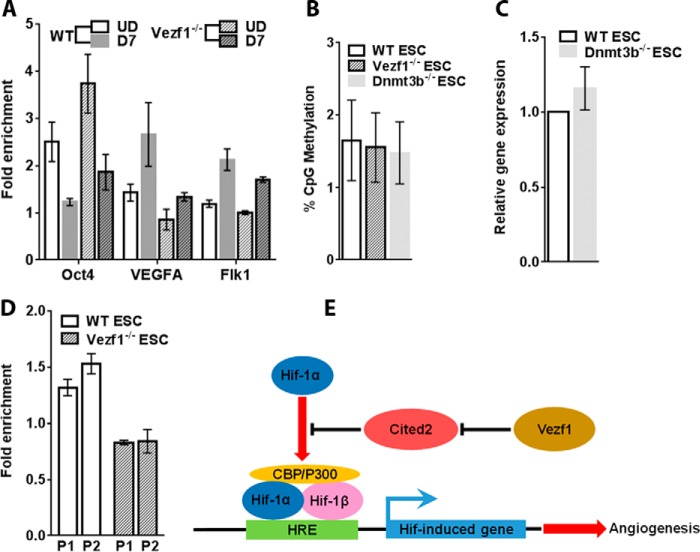
*A,* ChIP-qPCR of H3K27Ace showed an increase in the fold-enrichment of H3K27Ace at VEGF-A and Flk1 promoters in WT ECs that were reduced in *Vezf1*^−/−^ ECs. At the Oct4 promoter, post-differentiation, deacetylation of H3K27 is accompanied by the loss of gene expression. Therefore, it serves as a negative control. *B,* DNA methylation was analyzed at *Cited2* CpGi using bisulfite sequencing in undifferentiated ESCs. The average percent methylation of 27 CpG sites in the CpGi was plotted. *C,* gene expression analysis of Cited2 in WT and Dnmt3b^−/−^ ESCs. There was no significant difference in *Cited2* expression levels. *D,* ChIP-qPCR using anti-Vezf1 antibody showed an increase in the fold-enrichment at *Cited2* promoter in WT ESCs. Fold-enrichment below 1 in *Vezf1*^−/−^ ESCs indicates absence of binding, and was used as negative control. The data in each *bar graph* represents average and S.D. of 3 to 4 replicates. *E,* model showing the effect of Cited2 on Hif-1α-mediated regulation of pro-angiogenic genes. Vezf1 regulates the expression of Cited2 at basal levels in ESCs before the induction of endothelial differentiation. This allows the interaction of Hif-1α and P300 that activate the pro-angiogenic genes. In the absence of Vezf1, high expression of Cited2 sequesters P300 away from Hif-1α, thus inhibiting the activation of pro-angiogenic genes. *WT*, wildtype; *Vezf1*^−/−^, *Vezf1* knockout; *Dnmt3b*^−/−^, Dnmt3b knockout; *UD*, undifferentiated; *D7*, days post-differentiation; *P1* and *P2*, two primer pairs in *Cited2* promoter used in ChIP-qPCR.

In response to VEGF signaling, P300 acetyltransferase interacts with Hif-1α, which targets it to the HBS (HIF-1–binding element) of the promoters of angiogenesis-specific genes where it acetylates histone H3 at Lys^27^. To test the impact of *Cited2* expression on the activity of P300 histone acetyltransferase at *VEGF* and *Flk-1* promoters, we performed a chromatin immunoprecipitation (ChIP) assay using anti-histone H3K27Ace antibody. We observed an expected increase in the fold-enrichment of H3K27Ace at VEGF and Flk-1 promoters in WT-differentiated ECs compared with the undifferentiated ESCs. However, this increase was markedly reduced in the differentiated *Vezf1*^−/−^ ECs particularly at the *VEGF* promoter, which is a direct target of Hif-1α. As a control, we used the *Oct4* promoter where H3K27 acetylation is decreased post-differentiation in both the WT and *Vezf1*^−/−^ ESCs (Fig. 8*A*). Although *Flk-1* has an HBS in its promoter, some studies indicate that it is targeted by both Hif-1α and HIF-1β (40). These data support our hypothesis that reduced P300 activity at angiogenesis-specific gene promoters inhibits their complete activation during *Vezf1*^−/−^ EC differentiation.

### Transcriptional regulation of Cited2 expression by Vezf1

*Vezf1*^−/−^ ESCs show a genome-wide loss of DNA methylation at several CpG islands (CpGi's) flanking tissue-specific genes and a significant decrease in the expression of the DNA methyltransferase, Dnmt3b (39). Cited2 is encoded by a relatively small gene, which has a large CpGi at its promoter and exon 1 (Fig. 1*C*). We therefore asked if the increase in *Cited2* expression in *Vezf1*^−/−^ ESCs could be due to loss of DNA methylation at its CpGi. To test the potential role of Dnmt3b in regulation of *Cited2* CpGi DNA methylation, genomic DNA from Dnmt3b^−/−^ ESCs, *Vezf1*^−/−^ ESCs, and WT ESCs was extracted to quantify DNA methylation using bisulfite sequencing. Our data show very low CpG methylation at *Cited2* CpGi in WT ESCs that did not change in *Vezf1*^−/−^ and Dnmt3b^−/−^ ESCs (Fig. 8*B*). *Cited2* gene expression also showed no change in Dnmt3b^−/−^ compared with that in WT ESCs (Fig. 8*C*). These data confirm that expression of *Cited2* is not regulated by Dnmt3b or changes in DNA methylation at its CpGi and support the direct role of Vezf1 in regulating *Cited2* expression. Therefore, we investigated the binding of Vezf1 near the *Cited2* promoter by ChIP assay using custom-made rabbit polyclonal anti-Vezf1 antibody, which was previously characterized and used in ChIP studies in ESCs (12, 34). We observed a high relative enrichment of Vezf1 at the *Cited2* promoter in WT ESCs (Fig. 8*D*). These data suggest a direct regulation of *Cited2* expression by Vezf1 through its binding at the promoter-associated CpGi.

Taken together our *in vitro* differentiation experiments show that the aberrant high expression of *Cited2* in *Vezf1*^−/−^ ESCs suppresses their angiogenic potential by sequestering P300/CBP away from the pioneer transcription factor Hif-1α. This abbreviates the promoter activation of the downstream pro-angiogenic genes (Fig. 8*E*). These findings suggest that Vezf1 regulates angiogenesis by fine-tuning the level of anti-angiogenic factor Cited2.

## Discussion

The transcription factor Vezf1 is highly expressed in vascular endothelium and its role in vascular development has been observed by several earlier studies (28, 34, 36, 44, 45). For example, recent studies have shown that a small molecule inhibitor of Vezf1, Vec6, prevents wound healing and angiogenesis (30). Although the function of Vezf1 has been suggested through its role as a transcriptional activator of some genes that are known to promote angiogenesis, previous studies were performed using semi-quantitative RT-PCR to measure the expression of pro-angiogenic genes in *Vezf1*^−/−^ embryos. These data showed no change in the expression of pro-angiogenic factors compared with WT embryos (28). Unlike the previous study (28), we used quantitative RT-PCR to measure gene expression changes and our data show about 2–3-fold lower expression of several pro-angiogenic genes, including *CD31*, *Tie2*, *VEGF-A*, and its receptors *Flk-1* and *Flt-1* in the *in vitro* derived *Vezf1*^−/−^ ECs. The defective EC differentiation of *Vezf1*^−/−^ ESCs was also supported by impaired morphological changes associated with EC differentiation. We further show that the expression of some of these genes can be largely rescued by down-regulating *Cited2*, which is aberrantly overexpressed in *Vezf1*^−/−^ ESCs. Our data support the previously suggested role of Vezf1 in angiogenesis, however, through a different mechanism. In contrast to its previously predicted role as a transcriptional activator, our data show that Vezf1 restricts the expression of the anti-angiogenic gene *Cited2* to basal levels, ensuring a balanced gene expression during angiogenesis. Our data also emphasize that small but quantifiable changes in gene expression of developmental transcription factors and regulators can have profound effect on cell differentiation.

Cited2 (Mrg1/p35srj) belongs to a family of transactivators that lack direct DNA binding but contain glutamic acid/aspartic acid (ED)-rich tail, which interacts with P300/CBP acetyltransferase. Whereas, on one hand, Cited2 competes with Hif-1α to interact with P300/CBP, *Cited2* promoter has HIF1-binding sites and its expression is up-regulated in hypoxia by Hif-1α. Therefore, Cited2 participates in a negative-feedback loop with Hif-1α in which Cited2 accumulates during hypoxia. During restoration of normaxia, it will inhibit Hif-1α activity and prevent hypervascularization. An anomalous high expression of *Cited2* at the onset of angiogenesis could interfere with Hif-1α-mediated activation of pro-angiogenic genes, consequently the expression of *Cited2* must be tightly controlled. We propose that in undifferentiated ESCs, Vezf1 modulates the expression of *Cited2*, thus enabling Hif-1α–mediated gene activation during angiogenesis. This mechanism is supported by our data showing that overexpression of *Cited2* causes defective tube formation by *Vezf1*^−/−^ ECs when there is no difference in Hif-1α expression between the WT and *Vezf1*^−/−^ cells. *Cited2* is expressed throughout early embryogenesis and its expression in ESCs is critical for pluripotency, and appropriate differentiation (13, 43). Our data showing absence of rescue in a *Vezf1*^−/sh^ (3-3) cell line, which has *Cited2* expression significantly lower than WT ESCs, supports the role of the basal expression of *Cited2* in maintenance of pluripotency and differentiation potential of ESCs. Vezf1-mediated regulation of *Cited2* expression is also potentially relevant during adult angiogenesis and wound healing where Vezf1 could down-regulate or maintain low *Cited2* expression in the ECs. This prediction is supported by our observation from a published microarray analysis of *Vezf1*-silenced BVEC's, listing *Cited2* among the up-regulated genes (30). This study also showed that loss of Vezf1 causes inhibition of wound healing and blood vessel formation (30).

Based on the previously characterized role of Vezf1 as an insulator binding protein, it is highly plausible that Vezf1 insulates its target promoters from interaction with nonspecific enhancers, and in the case of *Cited2* from the enhancers in the downstream gene, β-*taxilin* (*Txlnb*). The insulator function of Vezf1 is supported by our published ChIP-SEQ studies showing that a significant number of Vezf1-binding sites are adjacent to insulator protein, CTCF. Vezf1 shows widespread binding at CpGi's present in the regulatory elements of genes including promoters, enhancers, and insulators (32, 38). By using *in vitro* EC differentiation as a developmental model system, it will be important to explore the regulatory potential of Vezf1-mediated insulator function in modulating gene expression during development.

## Experimental procedures

### Embryonic stem cell culture

Undifferentiated WT, Dnmt3b^−/−^, and *Vezf1*^−/−^ ESCs were cultured in Dulbecco's modified Eagle's medium containing 15% ESC qualified fetal bovine serum (Millipore), supplemented with nonessential amino acids, glutamine, 1000 units/ml of leukemia inhibitory factor (LIF) (ESGRO; Chemicon International), and 50 μm β-mercaptoethanol. Cells were cultured on 0.1% gelatin for one passage before switching to differentiation conditions.

### Endothelial lineage differentiation

ESCs were plated at a density of 3 × 10^3^ cells/cm^2^ on gelatinized plates in ESC medium with LIF and incubated overnight to attach. The next day the media was removed and cells were washed with PBS. Differentiation was induced by adding ESC medium without LIF and VEGF-A_165_ (R&D Systems) at 20–60 ng/μl. VEGF-A_165_ was supplemented to the culture every alternate day for 10 days to drive differentiation into endothelial lineage. Cell morphology was monitored using phase-contrast microscopy and pluripotency was monitored by alkaline phosphatase staining.

### Tube-formation assay

The ECs on D10 post-differentiation were collected by trypsinization and counted using Bio-Rad Cell Counter. The tube-formation assay was performed by plating 2 × 10^5^ ECs on a 24-well plate coated with VEGF supplemented Matrigel (BD Biosciences) according to the manufacturer's protocol (46, 47). The cells were incubated at 37 °C for 3–18 h. Tubing was scored using images from phase-contrast microscopy (29). The length of the tubes was measured by ImageJ software.

### Transfection and generation of stable ESC lines

The lentivirus construct pLKO.1 carrying shRNA specific for Cited2 was purchased from Dharmacon. The recombinant lentivirus containing Cited2 shRNA was packaged using Vira Power (Fisher Thermo Scientific) in 293FT cells using the manufacturer's protocol. For shRNA-mediated depletion of Cited2, WT, and *Vezf1*^−/−^ ESCs were transfected by lentivirus at a multiplicity of infection of 2 followed by selection of transgenic lines with stably integrated lentivirus construct using 3 μg/ml of puromycin.

### Gene expression by quantitative RT-PCR and Western blotting

RNA from cells was purified using TRIzol (Invitrogen, 15596026) according to the manufacturer's protocol. Genomic DNA contamination was removed by DNase (Roche Applied Science, 04716728001) treatment at 37 °C overnight. Quantitative RT-PCR was performed for equal amounts of RNA by using Verso One-Step kit (Thermo Scientific, AB-4104A). The data were analyzed and gene expression was normalized to *Gapdh* expression. The change in expression is represented either as normalized gene expression or as relative gene expression that is changed relative to expression in the undifferentiated cells, set to 1. See Table S1 for primers used. Western blot analysis was performed using commercially available antibodies anti-Cited2 (ab108345, Abcam) and anti-Gapdh (sc47724, Santa Cruz) according to the manufacturers' recommendation.

### DNA-methylation assay

DNA-methylation assay was performed by bisulfite sequencing (Bis-SEQ). Bisulfite sequencing was performed using the EpiTect Fast Bisulfite Conversion Kit (Qiagen, 59802). Genomic DNA was purified from WT, *Vezf1*^−/−^, and Dnmt3b^−/−^ ESCs. 1 μg of genomic DNA was used and bisulfite-converted DNA was amplified using nested primers and *Taq* polymerase (New England Biolabs, M0267L). Products from the inner PCR were used to generate a library for high throughput sequencing using Wide-SEQ. The primers used are listed in Table S1.

### ChIP

ChIP was performed using cross-linked chromatin from WT and *Vezf1*^−/−^ ESCs using a previously published protocol (18). Briefly, cells were cross-linked for 5 min with 1% formaldehyde in buffer (0.1 m NaCl, 1 mm EDTA, 0.5 mm EGTA, and 50 mm HEPES, pH 8). Nuclei were isolated and chromatin was sheared to 0.5–1-kb fragments using a Covaris E210 device, according to the manufacturer's protocols. Antibodies (8 μg) were immobilized on Protein A/G magnetic beads (Life Technologies, 10002D and 10004D) by overnight incubation. The magnetic beads were washed to remove unconjugated antibody and mixed with 8 μg of sonicated chromatin. After an overnight incubation, magnetic beads were washed, and bound DNA was purified. DNA was quantified using PicoGreen (Life Technologies, P11495) in a NanoDrop 3300 fluorospectrometer. Quantitative PCR was then performed using equal amounts of IN (input) and IP (immunoprecipitated sample) DNA. Fold-enrichment was calculated as follows: *C_t_*(IN)–*C_t_*(IP) and the fold-change was calculated by using 2∧(*C_t_*(IN)–*C_t_*(IP)). The fold-enrichment of 1 or less indicates no binding. See Table S1 for primers used. The H3K27Ace antibody used is commercially available (39133, Active Motif) and anti-Vezf1 antibody is a previously characterized custom-made polyclonal antibody.

## Author contributions

L. A., M. H., Q. Y., and H. G. formal analysis; L. A., M. H., and A. B. N. validation; L. A., M. H., Q. Y., and A. B. N. investigation; L. A. and Q. Y. methodology; L. A., M. H., and H. G. writing-review and editing; M. H. visualization; M. H. and H. G. project administration; Q. Y. and H. G. writing-original draft; H. G. conceptualization; H. G. supervision; H. G. funding acquisition.

## Supplementary Material

Supporting Information

## Footnotes

4 The abbreviations used are:

EC: endothelial cell
VEGF: vascular endothelial growth factor
Cited2: Cbp/p300-interacting transactivator with Glu/Asp-rich carboxyl-terminal domain 2
CBP: cAMP-responsive element-binding protein
ESC: embryonic stem cell
Vezf1: vascular endothelial zinc finger 1
LIF: leukemia inhibitory factor
IN: input
IP: immunoprecipitated
qPCR: quantitative PCR
Gapdh: glyceraldehyde-3-phosphate dehydrogenase
D: days post-differentiation.
