# Supplementary material for: The transcription factor Vezf1 represses the expression of the antiangiogenic factor Cited2 in endothelial cells
Source: J Biol Chem. 2018 May 24;293(28):11109–18. doi: 10.1074/jbc.RA118.002911 (PMC6052231; doi:10.1074/jbc.RA118.002911)
Supplement: Supporting Information [file supp_293_28_11109__index.html]

The transcription factor Vezf1 represses the expression of the antiangiogenic factor Cited2 in endothelial cells — Cited2 causes angiogenesis defects in Vezf1 null cells — The transcription factor Vezf1 represses the expression of the antiangiogenic factor Cited2 in endothelial cells — Cited2 causes angiogenesis defects in Vezf1 null cells — Supporting Information 

# The transcription factor Vezf1 represses the expression of the antiangiogenic factor Cited2 in endothelial cells

## Supporting Information

- The transcription factor Vezf1 represses the expression of the antiangiogenic factor Cited2 in endothelial cells - List of materials: Supplementary Figure S1 Table S1
