# Supplementary material for: The transcription factor Vezf1 represses the expression of the antiangiogenic factor Cited2 in endothelial cells
Source: J Biol Chem. 2018 May 24;293(28):11109–18. doi: 10.1074/jbc.RA118.002911 (PMC6052231; doi:10.1074/jbc.RA118.002911)
Supplement: Supporting Information [file supp_RA118.002911_136674_2_supp_140936_p8z452.pdf]

SUPPLEMENTARY INFORMATION

The transcription factor Vezf1 represses the expression of the antiangiogenic factor Cited2 in endothelial cells

Lama AlAbdi[Δ], Ming He[Δ], Qianyi Yang[#], Allison B. Norvil and Humaira Gowher[1, 2]*

List of materials:

Supplementary Figure S1

Table S1

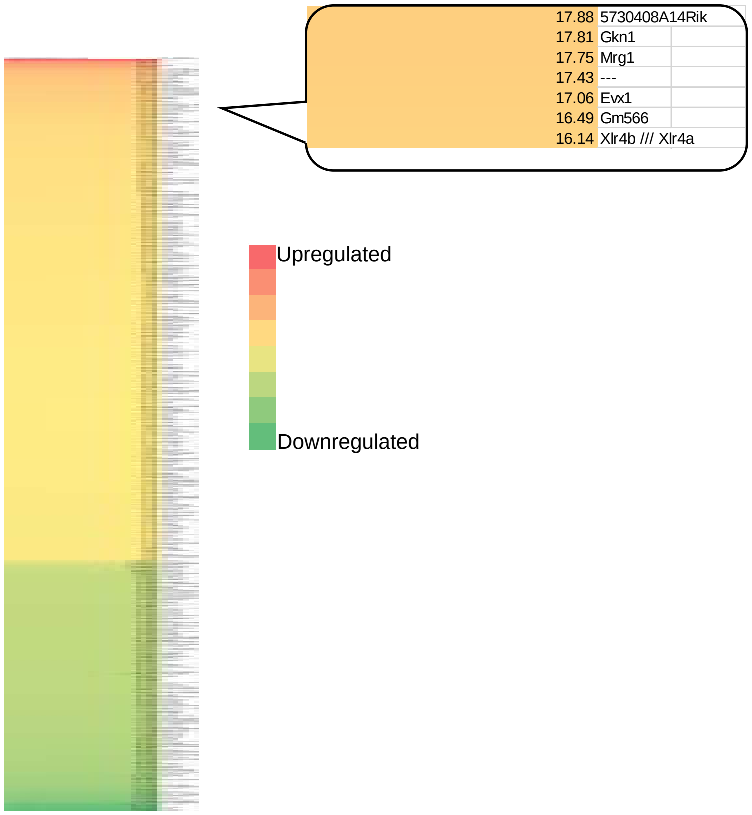

Figure S1. RNA was purified from WT and Vezf1$^{-/-}$ ESCs and oligonucleotide analysis using the Affymetrix platform, were done according to the manufacturer's instructions (Affymetrix, Santa Clara, CA). The full protocol can be found at http://www.affymetrix.com/support/technical/manual/expression_manual.affx. Microarray analysis was performed at the NIDDK microarray core facility, NIH, Bethesda MD. Briefly, RNA extraction from cultured ESCs was performed using the TriZol (Invitrogen, Carlsbad, CA) and RNeasy (Qiagen, Valencia, CA). Twenty micrograms of purified RNA was used as a template for double-stranded cDNA synthesis. It was then hybridized to Affymetrix mouse genome arrays. Affymetrix probe sets with "Absent Calls" were eliminated from further statistical analysis. An ANOVA model with log (base 2) of the Affymetrix signal as a response was fitted for each one of the remaining Affymetrix probe sets. The signals from Vezf1$^{-/-}$ RNA were compared to that of WT and fold change was calculated using ANOVA4. The upper threshold of the p-value used for measuring the significance was set to 10^{-2}. Genes whose expression increased or decreased by more than 5 fold in Vezf1$^{-/-}$ ESC compared to WT were sorted (p-value ranging from 10^{-8} to 10^{-3}). Two probes for Mrg1/Cited2 showed 17 and 5 fold increase in expression in the Vezf1$^{-/-}$ ESCs with p-values at 10^{-5} and 10^{-3} respectively.

Table S1

For Bisulfite PCR:	***Primer***
Cited2proBS out F	TTGTAGAGGGTAGGGTAGATTATTA
Cited2proBS out R	CCAAACAACTACCAACAATAAACTATATTT
Cited2proBS In F	GGGTGGGGAGATTTAGTTAGAA
Cited2proBS In R	AACTTTAACCACTATTAATATAAAAATCTCAAAA
**For RT-qPCR:**	**Primer**
Cited2 RT F2	GAAGGACTGGAAATGGCAGACCATATGAT
Cited2 RT R2	TGGTGCAGCCCGTTGGTG
Oct3/4 RT F	TCTTTCCACCAGGCCCCCGGCTC
Oct3/4 RT R	TGCGGGCGGACATGGGGAGATCC
Gapdh RT F	CAAAATGGTGAAGGTCGGTGTGAA
Gapdh RT R	CAACAATCTCCACTTTGCCACTG
VEGFA RT F	AAGGAGAGCAGAAGTCCCATGA
VEGFA RT R	CTCAATTGGACGGCAGTAGCT
Flk1 RT F	TCTGTGGTTCTGCGTGGAGA
Flk1 RT R	GTATCATTTCCAACCACCC
CD31 RT F	TACTGCAGGCATCGGCAAA
CD31 RT R	GCATTTCGCACACCTGGAT
Tie2 RT F	AAGACATACGTGAACACCACACT
Tie2 RT R	ACTCTAGAGTCAGAACACACTGCAGAT
Hif-1α F	TCCCAGCATCTATGGCTTGA
Hif-1α R	GCTGTAACAGGAGAAGGGGG
Flt1 F	GGGCTTCAGACAGACAGGAC
Flt1 R	TGTCTTGTGGAAGAGCTCACT
Dnmt3b RT F	ATGGCTTCAAAGAATGATAAGCTCG
Dnmt3b RT R	*TCTGCACTTTCTTTAACTTTGCTGT*
**For ChIP:**	**Primer**
Cited2 pro 1 F	TTCTTCATTACAAACCCGTTTGCTTC
Cited2 pro 1 R	TCTGCTGTTGCCCTTGATCC
Cited2 pro 2 F	CTAGGTAAGCCGGGTGAGCG
Cited2 pro 2 R	GCTAGTCCTCCCGGCCAC
Oct4 pro F	CCCGTCCTAAGGGTTGTCCT
Oct4 pro R	GTGGGGGTGGGAGAAACTG
VEGFA pro F	TCCGCTGAATAGTCTGCCTTG
VEGFA pro R	GTTACCGGTGAGAAGCGCA
Flk-1 pro F	ATCGCTGTTTCCAGGCTACG
Flk-1 pro R	GCGCTGCGACTTTGATACAC
